# One-Pot synthesis, characterization and adsorption studies of amine-functionalized magnetite nanoparticles for removal of Cr (VI) and Ni (II) ions from aqueous solution: kinetic, isotherm and thermodynamic studies

**DOI:** 10.1186/s40201-016-0252-0

**Published:** 2016-07-26

**Authors:** Abbas Norouzian Baghani, Amir Hossein Mahvi, Mitra Gholami, Noushin Rastkari, Mahdieh Delikhoon

**Affiliations:** 1 Center for Water Quality Research, Institute for Environmental Research (IER), Tehran University of Medical Sciences, Tehran, Iran; 2 Department of Environmental Health Engineering, School of Public Health Science, Tehran University of Medical Sciences, Tehran, Iran; 3 Center for Solid Waste Research, Institute for Environmental Research (IER), Tehran University of Medical Sciences, Tehran, Iran; 4 Research Center for Environmental Health Technology, Iran University of Medical Sciences, Tehran, Iran; 5 Environmental Health Department, School of Public Health, Iran University of Medical Sciences, Tehran, Iran; 6 Center for Air Pollution Research (CAPR), Institute for Environmental Research (IER), Tehran University of Medical Sciences, Tehran, 1417613151 Iran; 7 Environmental Health Department, School of Public Health, Shiraz University of Medical Sciences, Shiraz, Iran

**Keywords:** Fe_3_O_4_-NH_2_, Heavy metal ions, Amino-functionalized, 1, 6 hexanediamine, Thermodynamics, Adsorption/desorption

## Abstract

**Background:**

Discharge of heavy metals such as hexavalent chromium (Cr (VI)) and nickel (Ni (II)) into aquatic ecosystems is a matter of concern in wastewater treatment due to their harmful effects on humans. In this paper, removal of Cr (VI) and Ni (II) ions from aqueous solution was investigated using an amino-functionalized magnetic Nano-adsorbent (Fe_3_O_4_-NH_2_).

**Methods:**

An amino-functionalized magnetic Nano-adsorbent (Fe_3_O_4_-NH_2_) was synthesized by compositing Fe_3_O_4_ with 1, 6-hexanediamine for removal of Cr (VI) and Ni (II) ions from aqueous solution. The adsorbent was characterized by Scanning Electron Microscope (SEM), Transmission Electron Microscopy (TEM), powder X-Ray Diffraction (XRD), and Vibrating Sample Magnetometry (VSM). Also, the effects of various operational parameters were studied.

**Results:**

According to our finding, Fe_3_O_4_-NH_2_ could be simply separated from aqueous solution with an external magnetic field at 30 s. The experimental data for the adsorption of Cr (VI) and Ni (II) ions revealed that the process followed the Langmuir isotherm and the maximum adsorption capacity was 232.51 mg g^−1^ for Cr (VI) at pH = 3 and 222.12 mg g^−1^ and for Ni(II) at pH = 6 at 298 °K. Besides, the kinetic data indicated that the results fitted with the pseudo-second-order model (R^2^: 0.9871 and 0.9947 % for Cr (VI) and Ni (II), respectively. The results of thermodynamic study indicated that: standard free energy changes (ΔG^ɵ^), standard enthalpy change (ΔH^ɵ^), and standard entropy change (ΔS^ɵ^) were respectively −3.28, 137.1, and 26.91 kJ mol^−1^ for Cr (VI) and −6.8433, 116.7, and 31.02 kJ mol^−1^ for Ni (II). The adsorption/desorption cycles of Fe_3_O_4_-NH_2_ indicated that it could be used for five times.

**Conclusions:**

The selected metals’ sorption was achieved mainly via electrostatic attraction and coordination interactions. In fact, Fe_3_O_4_-NH_2_ could be removed more than 96 % for both Cr (VI) and Ni (II) ions from aqueous solution and actual wastewater.

## Background

Discharge of heavy metals into aquatic ecosystems is a matter of concern in wastewater treatment due to their harmful effects on humans even at low concentrations [1, 2]. Among heavy metals, Cr (VI) is among the toxic elements that may enter the environment due to effluent discharge by some industries, such as tanning, textile, wood preservations, paint, metal and mineral processing, pulp, and paper industries [3, 4]. Evidence has shown that these elements can be carcinogenic and mutagenic to living organisms [5]. Nickel is also another heavy metal used in different industries, such as porcelain enameling, electroplating, storage batteries, dying, steel manufacturing, and pigment industries. The acceptance tolerance of nickel has been reported to be 0.01 mgL^−1^ and 2.0 mgL^−1^ in drinking water and industrial wastewater, respectively [6]. Due to the problems remarked above, some effective wastewater treatment approaches have to be employed for Cr (VI) and Ni (II) removal. Up to now, many methods have been used in this regard, including chemical precipitation, ion exchange, membrane technologies, coagulation, electrocoagulation, reduction, bio sorption, filtration, adsorption, reverse osmosis, foam flotation, granular ferric hydroxide, electrolysis, and surface adsorption [7–11]. Most of these methods have economic and technical disadvantages and could not achieve the discharge standards. Yet, adsorption is an effective and flexible method, generating high-quality treated effluent [12]. Until now, many adsorbents have been grown, including maple sawdust, walnut, hazelnut, almond shell [2], carbon nanotubes [13], amino-functionalized polyacrylic acid (PAA) [14], and Lewatit FO36 Nano [15]. However, in many cases, these materials do not have the sufficient adsorption efficiency because of not having enough active surface sites. Furthermore, these materials have a lot of problems, including high cost, difficulty in separation, desorption, and regeneration of adsorbents, and secondary wastes. Therefore, new materials, such as various functional groups, including amide, amino groups, and carboxyl, are to develop new adsorbents that have high selectivity toward toxic metals [16–18]. In this respect, amino-groups have attracted more attention as chelation sites due to their large specific surface areas. Thus, amino-groups are capable of adsorbing a number of metal anions and cations from aqueous solution [19].

As described above, due to the high specific surface area created through grafting of appropriate organic amino-groups on inorganic magnetic Fe_3_O_4_ particles, with strong magnetic properties, low toxicity, and easy separation, it could be used as a sorbent for removing heavy metals [18, 20]. Another advantage is that it is useful for recovery or reuse of the magnetite nanoparticles modified with amino-groups [17, 21].

In this study, we prepared a novel amino-functionalized magnetic Nano-adsorbent (Fe_3_O_4_-NH_2_) developed by grafting amino-groups onto the surfaces of Fe_3_O_4_ nanoparticles and used nanocompostie as the adsorbent for removal of Cr (VI) and Ni (II) from aqueous solution. The adsorbent was characterized by Transmission Electron Microscopy (TEM), powder X-Ray Diffraction (XRD), Vibrating Sample Magnetometry (VSM), Scanning Electron Microscope (SEM), and zeta-potential measurement. The effects of pH, initial concentrations of Cr (VI) and Ni (II), adsorption kinetics, thermodynamics, and adsorption isotherm were studied, as well.

## Methods

### Chemicals

Anhydrous sodium acetate, iron (III) chloridehexahydrat (FeCl_3_ · 6H_2_O), potassium dichromate, ethanol, 1,6-hexanediamine, ethylene glycol, nickel (II) chloride hexahydrat (NiCl_2_.6H_2_O), sodium hydroxide, hydrogen chloride, which were of analytical grade, were purchased from Merck, Germany and were used without further purification. Potassium dichromate (99 %) and nickel (II) chloride hexahydrat (99 %) were used for preparation of Cr (VI) and Ni (II) solution. Additionally, doubly distilled deionized water was used throughout the work.

### Synthesis of amino-functionalized magnetic Nano-adsorbent (Fe_3_O_4_-NH_2_) by one-pot synthesis

Amino-functionalized magnetic Nano-adsorbent (Fe_3_O_4_-NH_2_) was prepared according to hydrothermal reduction method. In doing so, a solution of 1, 6-hexanediamine (13 g), anhydrous sodium acetate (4.0 g), and FeCl_3_ · 6H_2_O as a single Fe ion source (2.0 g) was added to ethylene glycol (80 mL). The above mixture was stirred at 50 °C under vigorous stirring for 30 min. Then, this solution was heated at 198 °C in a Teflonlined autoclave for 6 h. Thereafter, the mixture was cooled down to room temperature. The magnetite nanoparticles were collected with a magnet and were then washed with water and ethanol (3 times) to effectively remove the solvent and unbound 1, 6-hexanediamine. Finally, the amino-functionalized magnetic Nano-adsorbent (Fe_3_O_4_-NH_2_) was dried in a vacuum oven at 50 °C before characterization and application [22]. The size and morphology of the Fe_3_O_4_-NH_2_ were showed by SEM (Holland, company: Philips). Besides, the magnetic property (M–H loop) of the typical magnetic nanoparticles bound with 1, 6-hexadiamine at 25 °C was characterized by VSM (MDKFD, Iran). The crystal structure and phase purity of Fe_3_O_4_-NH_2_ were also examined by XRD (Philips, Holland) using Cu Kα radiation (λ = 0.1541 nm) at 2^θ^, 30 kV, and 30 mA. Finally, the TEM image of Fe_3_O_4_-NH_2_ was examined using TEM, Model EM10C-100KV (Zeiss, Germany).

#### Adsorption experiments

The absorption experiments were conducted in 1000 ml Erlenmeyer flasks containing 50 ml Ni (II) and Cr (VI) solutions at 5 to 100 mg L^−1^ concentrations and 0.05 g of Fe_3_O_4_-NH_2._ The mixtures were stirred (200 rpm) at room temperature from 10 to 90 min. After adsorption, Fe_3_O_4_-NH_2_ with adsorbed Cr (VI) and Ni (II) was separated from the solution under the external magnetic field. The concentrations of Cr (VI) and Ni (II) ions in the solutions were measured by an Inductive Coupled Plasma (ICP-OES, Spectro arcos, Germany (Company: SPECTRO)).

In order to determine the effects of various factors, the experiments were performed at different Fe_3_O_4_-NH_2_ doses (0.1 to 0.3 g/L), initial concentrations of Cr (VI) and Ni (II) (5 to 100 mg/L), and temperatures (298.15 to 338.15 °K). Besides, each experiment was carried out in duplicate. The removal of Cr (VI) and Ni (II) by Fe_3_O_4_-NH_2_ and removal efficiency have been figured by equations in Table 1 [18].

**Table 1:** The kinetic, isotherm, and thermodynamic equations used for adsorption of Cr (VI) and Ni (II) onto Fe_3_O_4_-NH_2_

Kinetic models	Isotherm equations	Thermodynamic equations	Removal efficiency and equilibrium adsorption capacity	Ref.
Pseudo-first-order ln(*q*_*e*_ − *q*_*t*_) = *lnq*_*e*_ − *k*_1_ *t*	Freundlich isotherm $ln{q}_e= ln{K}_F+\frac{1}{n} \ln Ce$	Van^,^ t Hoff *ΔG* ^*θ*^ = − *RT* *lnK*	equilibrium adsorption capacity ${q}_e=\frac{\left(C0-Ce\right)V}{m}$	[31–38]
Pseudo-second-order $\frac{\mathrm{t}}{\mathrm{q}\mathrm{t}}=\frac{1}{{\mathrm{K}}_2{\mathrm{q}}_{\mathrm{e}.\mathrm{c}}^2}+\frac{\mathrm{t}}{{\mathrm{q}}_{\mathrm{e}.\mathrm{c}}}$	Langmuir adsorption model $\frac{C_e}{q_e}=\frac{1}{K_l{q}_m}+\frac{C_e}{q_m}$	Free energy of adsorption $\mathrm{L}\mathrm{n}K=-\frac{\varDelta \mathrm{H}\uptheta}{RT}+\frac{\varDelta S\theta }{R}$	Removal efficiency $\left(\%\right)=\frac{\left({C}_i-{C}_o\right)}{C_i}\times 100$	[27, 31, 33–37, 39]
	Separation factor (R_L_) *R*_*L*_ = 1/(1 + *K*_*L*_ *C*_*o*_)			[18, 25, 26]
qe (mg/g), K_1_ (1/min), K_2_ (g/mgmin), qm (mg/g), K_F_ [(mg g^−1^) (mgL^−1^) n], Kp (mg/g min^-0.5^), C_0_ (mg/L), ΔH^ɵ^ (kJ/mol), ΔS^ɵ^ (J/mol.K), T (K), R (8.314 J/mol.K),V (mL), m (mg)				

## Results and Discussion

### Characterization of Fe_3_O_4_-NH_2_

**Fig. 1:**
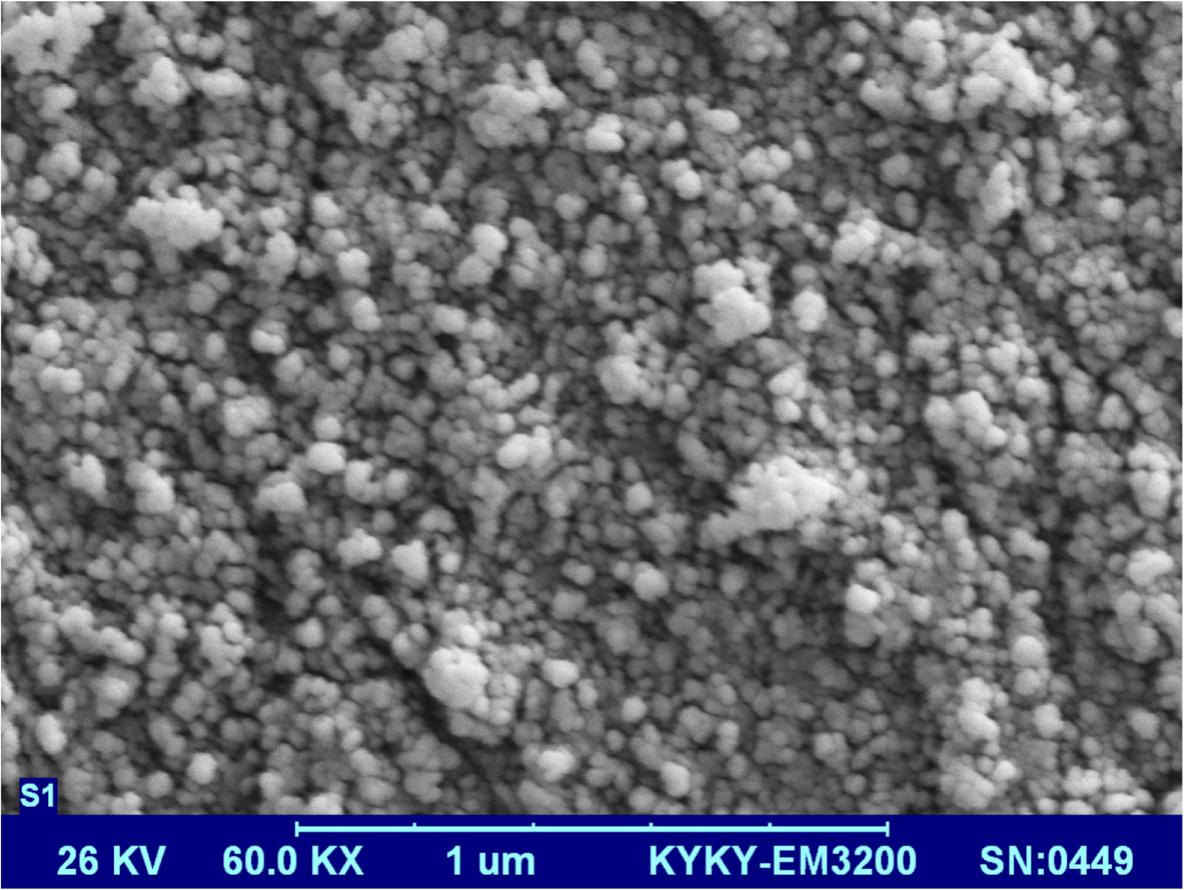
SEM image of Fe_3_O_4_ -NH_2_

The SEM, VSM, XRD, and TEM of Fe_3_O_4_-NH_2_ were recorded. The SEM of Fe_3_O_4_-NH_2_ image has been shown in Fig. 1. Based on the results, the SEM image indicated that the size of Fe_3_O_4_-NH_2_ was much smaller than that of naked particles, confirming the coating of 1, 6 hexanediamine [18].

**Fig. 2:**
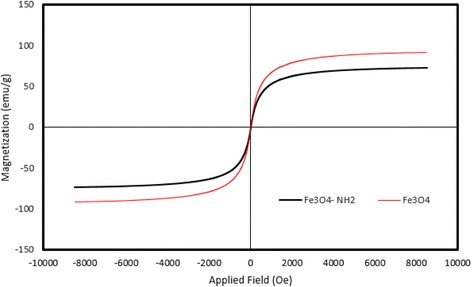
VSM magnetization curves of Fe_3_O_4_-NH_2_ and Fe_3_O_4_

**Fig. 3:**
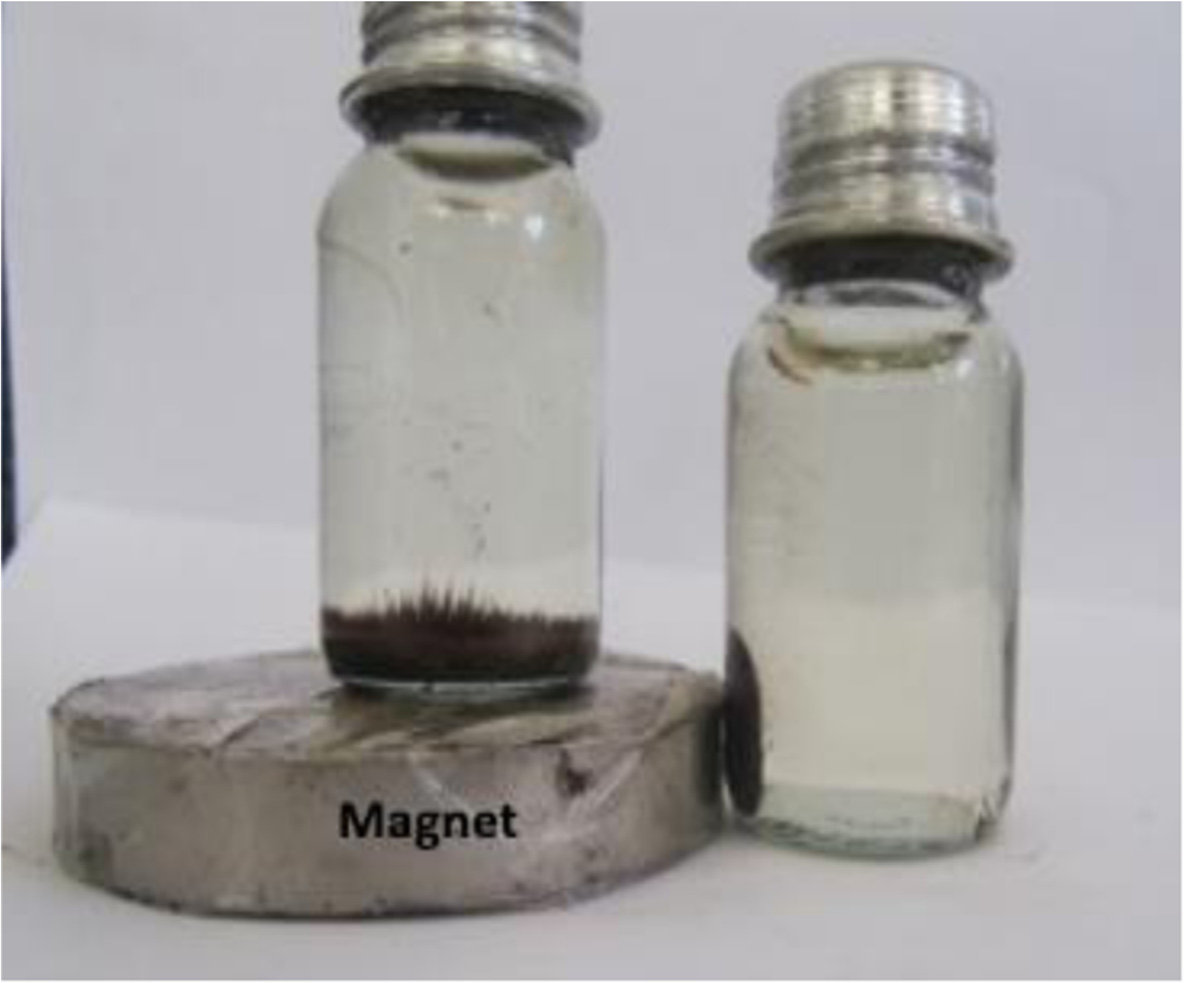
Demonstration of magnetic separation at 30 s

The magnetic hysteresis loops measured at room temperature has been illustrated in Fig. 2. The M–H curves showed that Fe_3_O_4_ and Fe_3_O_4_-NH_2_ were essentially super-paramagnetic. Fe_3_O_4_-NH_2_ and Fe_3_O_4_ have a magnetization saturation value of 73.25 and 91.57 emu g^−1^, respectively. According to Fig. 3, the magnetic Fe_3_O_4_-NH_2_ was dispersed in water. In addition, it could be collected by external magnetic field and be re-dispersed through slight shaking, making the solid and liquid phases separate easily.

**Fig. 4:**
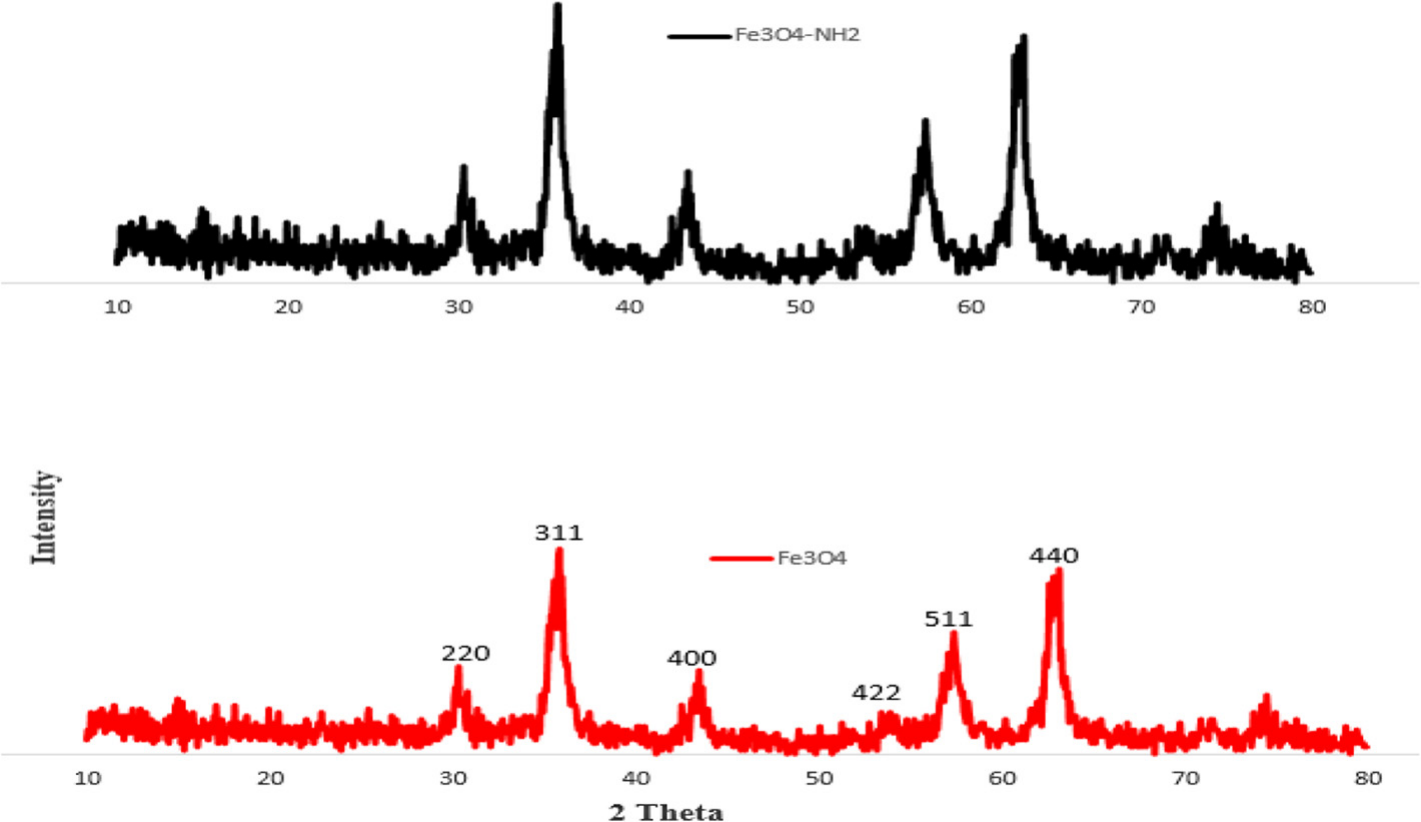
XRD for Fe_3_O_4_-NH_2_ and Fe_3_O_4_

The XRD patterns of Fe_3_O_4_-NH_2_ have been shown in Fig. 4. In this study, the crystal structure and phase purity of Fe_3_O_4_-NH_2_ were examined by XRD using a Cu Kd radiation (λ = 0.1541 nm) at 2^θ^ of 30.1°, 35.5°, 43.1°, 53.4°, 57.0°, and 62.6° corresponding to their indices; i.e., 220, 311, 400, 422, 511, and 440, at 30 kV and 30 mA. The particle size was obtained via XRD analysis through Debye-Sherrer’s formula [23]: D = K λ/β COS θ.

Where λ is the wavelength of the X-rays, θ is the diffraction angle, and β is the corrected full width. The result of size distribution demonstrated that the size of the prepared Fe_3_O_4_-NH_2_ was under 90 nm. Additionally, the sharp and strong peaks of the products revealed its appropriate crystallinity. Moreover, the six characteristic peaks of Fe_3_O_4_ showed that amino-groups did not cause any measureable alter in the phase property of Fe_3_O_4_ cores. Therefore, the amino-groups were fixed on the surface of Fe_3_O_4_ cores, making a core-shell structure. In other words, binding and amino-functionalization (NH_2_) occurred only on the surface of Fe_3_O_4_ cores to form a core–shell structure [22].

**Fig. 5:**
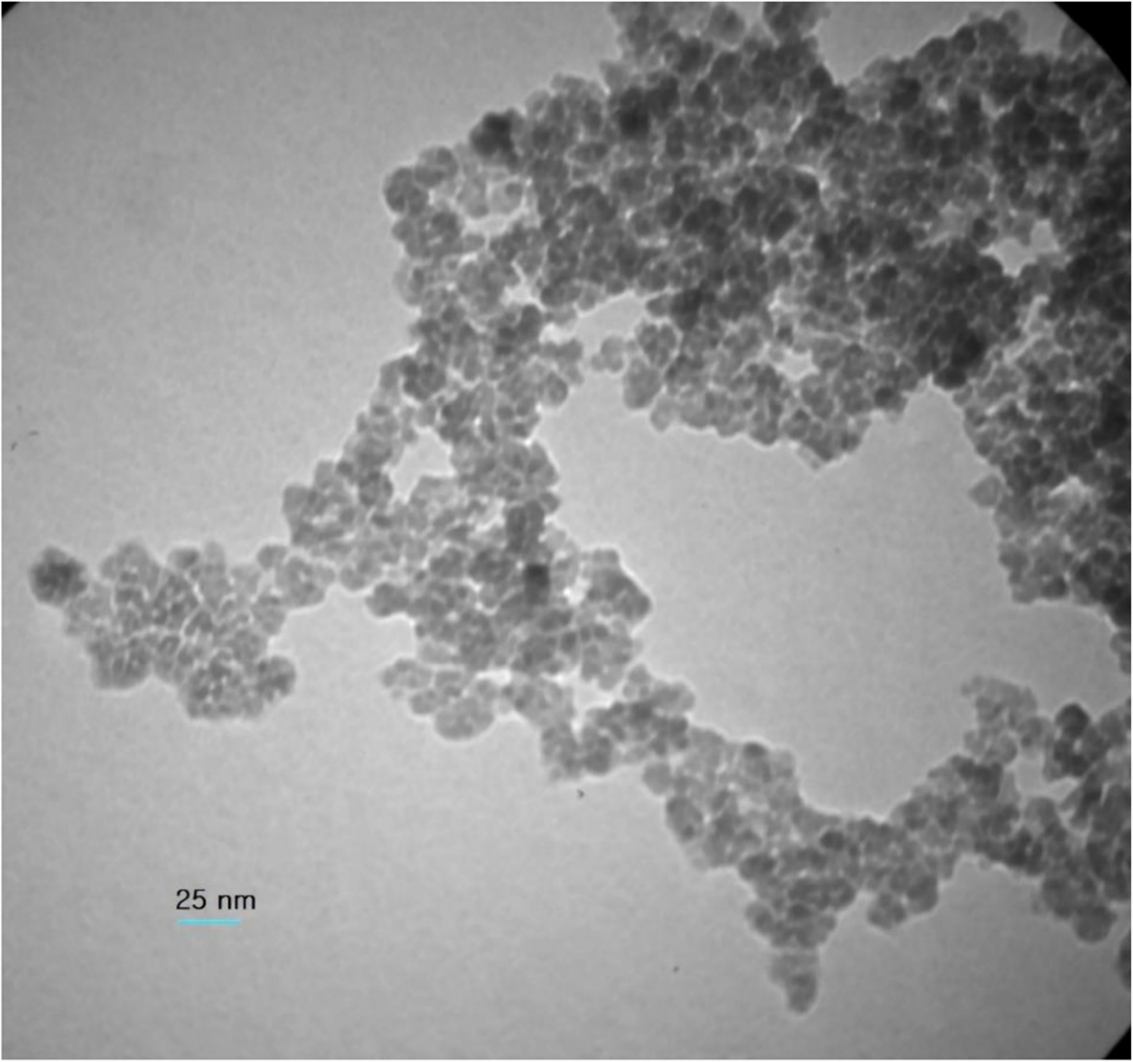
TEM image of Fe_3_O_4_-NH_2_

The TEM image of Fe_3_O_4_-NH_2_ has been shown in Fig. 5. Accordingly, Fe_3_O_4_-NH_2_ particles were multi-dispersed with an average diameter of around 25 nm. It has been reported that magnetic particles of less than 30 nm would show paramagnetism [24].

### The effect of initial concentration and pH on the adsorption properties and zeta potential analyses

**Fig. 6:**
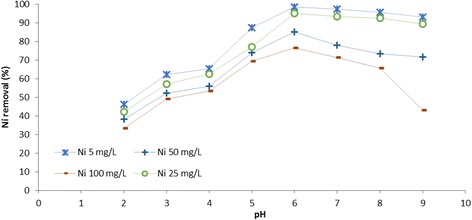
The effect of pH on the adsorption of Ni (II) onto Fe_3_O_4_-NH_2_ at different initial concentrations

**Fig. 7:**
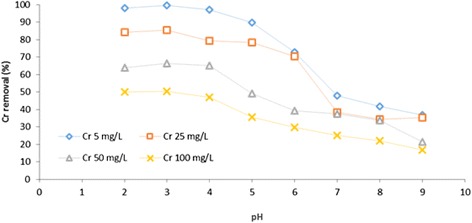
The effect of pH on the adsorption of Cr (VI) onto Fe_3_O_4_-NH_2_ at different initial concentrations

The effect of initial concentration on the adsorption properties was intensively studied for Fe_3_O_4_-NH_2_ by varying C_0_ of Cr (VI) and Ni (II) ions at 5, 25, 50, and 100 mg L^−1^. The results have been presented in Figs. 6 and 7. Under corresponding pH values from 2.0 to 9.0, the adsorption efficiency of Cr (VI) and Ni (II) respectively decreased and increased with increase in the initial Cr (VI) and Ni (II) concentrations. Accordingly, the percentage of uptake of Cr (VI) and Ni (II) ions at the Fe_3_O_4_-NH_2_ concentration of 5 mg L^−1^ decreased from 98.02 to 36.85 % for Cr (VI) and increased from 46.21 to 93.03 % for Ni (II) with increasing the pH from 2.0 to 9.0. This can be justified by the fact that for a fixed adsorbent dosage, the total available adsorption sites would be relatively settled. Thus, increasing the initial Cr (VI) and Ni (II) concentrations led to a decrease in the adsorption percentage of the adsorbate [25].

To assess the effect of pH, the study was conducted from pH 2 to 9 for both Cr (VI) and Ni (II) ions. The maximum sorption was perceived at pH = 6 for Ni (II), but at pH = 3 for Cr (VI). The adsorption of Cr (VI) at lower pH levels was also observed in other magnetic materials, such as the mesoporous magnetic ɤ-Fe_2_O_3_ [26]. pH value affected the adsorption efficiency due to its influence on the amino-groups modified on the surface of Fe_3_O_4_-NH_2_. The plot of pH initial vs. pH final depicted that the pHzpc was 5.8 for Fe_3_O_4_–NH_2_. Hence, at pH >5.8, the surface charge of Fe_3_O_4_-NH_2_ was negative and the electrostatic interactions between the metal ions and the adsorbent enhanced. Considering Ni (II), the interaction between the adsorbents and the Ni (II) ions might be defined by Equations 1–5 [14, 27].

1 $$ \mathrm{R}-\mathrm{N}{\mathrm{H}}_2+{\mathrm{H}}^{+}\leftrightarrow \mathrm{R}-\mathrm{N}{\mathrm{H}}_3^{+}\left(\mathrm{amino}\kern0.5em \mathrm{protonate}\right) $$

2 $$ \mathrm{R}-\mathrm{N}{\mathrm{H}}_2+\mathrm{N}{\mathrm{i}}^{2+}\leftrightarrow \mathrm{R}-\mathrm{N}{\mathrm{H}}_2\mathrm{N}{\mathrm{i}}^{2+} $$

3 $$ \mathrm{R}-\mathrm{N}{\mathrm{H}}_2+\mathrm{O}{\mathrm{H}}^{-}\leftrightarrow \mathrm{R}-\mathrm{N}{\mathrm{H}}_2\mathrm{O}{\mathrm{H}}^{-} $$

4 $$ \mathrm{R}-\mathrm{N}{\mathrm{H}}_2\mathrm{O}{\mathrm{H}}^{-}+\mathrm{N}{\mathrm{i}}^{2+}\leftrightarrow \mathrm{R}-\mathrm{N}{\mathrm{H}}_2\mathrm{O}{\mathrm{H}}^{-}\dots \mathrm{N}{\mathrm{i}}^{2+} $$

5 $$ \mathrm{Or}\kern0.5em \mathrm{R}-\mathrm{N}{\mathrm{H}}_2\mathrm{O}{\mathrm{H}}^{-}+\mathrm{N}\mathrm{i}\mathrm{O}{\mathrm{H}}^{+}\leftrightarrow \mathrm{R}-\mathrm{N}{\mathrm{H}}_2\mathrm{O}{\mathrm{H}}^{-}\dots \mathrm{N}\mathrm{i}\mathrm{O}{\mathrm{H}}^{+} $$

The protonation/deprotonation reactions of the Fe_3_O_4_-NH_2_ amino-groups in the solution have been presented in Equation 1. Based on Equation 2, the ability of NH_2_ to be protonated was weakened at higher pH levels, resulting in more –NH_2_ on the surface of the adsorbent to coordinate with Ni (II). At higher pH levels, OH^−^ in the solution is competitively adsorbed by amino-groups (−NH_2_), and the electrostatic adsorption is prevailed gradually compared to coordination. Considering Cr (VI), a large number of H^+^ exists under acidic conditions (pH levels: 2–3.5), causing amino-groups (−NH_2_) to be protonated to NH^3+^ more easily and electrostatic attraction to occur between these two oppositely charged ions (Equation (6)) [14, 27].

6 $$ \mathrm{R}-\mathrm{N}{\mathrm{H}}^{3+}+\mathrm{H}\mathrm{C}\mathrm{r}{\mathrm{o}}_4^{-}\to \mathrm{R}-\mathrm{N}{\mathrm{H}}^{3+}\dots \dots \mathrm{H}\mathrm{C}\mathrm{r}{\mathrm{o}}_4^{-} $$

### Kinetic, equilibrium, and thermodynamic studies

Adsorption isotherms of Fe_3_O_4_-NH_2_ were gained at pH = 3 for Cr (VI) and pH = 6 for Ni (II) with the initial concentrations of 5 to 100 mg L^−1^. The relevant equations for kinetic, equilibrium, and thermodynamic studies have been shown in Table 1 [18]. Besides, the Langmuir and Freundlich parameters, correlation coefficients (R^2^), and separation factor (R_L_) for the adsorption of Cr (VI) and Ni (II) on Fe_3_O_4_-NH_2_ have been summarized in Table 2. The essential characteristics of the Langmuir isotherm can be expressed in terms of a dimensionless separation factor (R_L_). The plot of C_e_ vs. (C_e_/q_e_) for Fe_3_O_4_-NH_2_ gave a straight line with correlation coefficients of 0.998 and 0.994 for Cr (VI) and Ni (II), respectively. The q_max_ and K_L_ were derived from the slope and intercept of the line, respectively. The adsorption capacities (q_m_, mg/g) using the Langmuir isotherm equation were as follows: q_m_ Ni (II) (232.15) > q_m_ Cr (VI) (222.12). Considering the larger adsorption capacity of Fe_3_O_4_-NH_2_ attributed to the amino-groups modified on the surface of Fe_3_O_4_-NH_2_, the amino-groups played a very important role in the adsorption process of Cr (VI) and Ni (II) in aqueous solution. This implies that increasing the percentage of nitrogen in the Fe_3_O_4_-NH_2_ could increase the value of q_m_. The calculated R_L_ values for adsorption of Cr (VI) and Ni (II) were also 0.03–0.39 and 0.02–0.34, respectively, which fall between 0 and 1. Thus, the adsorption of Cr (VI) and Ni (II) onto NH_2_- Fe_3_O_4_ was favorable. However, the correlation coefficients (R^2^ > 0.99) and R_L_ (0 < R_L_ < 1) proved that the Langmuir isotherm fitted better for adsorption of Cr (VI) and Ni (II) on NH_2_- Fe_3_O_4_. On the other hand, the value of 1/n shows whether the adsorption is suitable for the Freundlich isotherm [18]. The value of 1/n reported in Table 2 was less than 1; hence, adsorption by the Freundlich model was unfavorable. Kinetics of the adsorption process is essential for aqueous solution since it gives essential information on the rate of adsorbate uptake on the adsorbent and controls the equilibrium time. The results presented in Table 2 indicated that the adsorption capacity of Fe_3_O_4_-NH_2_ for Cr (VI) and Ni (II) was high (q_m_ for Ni (II) = 232.51 mg/g^−1^ at pH = 6 and q_m_ for Cr (VI) = 222.12 mg/g^−1^ at pH = 3) compared to other adsorbents. Afkhami et al. also reported that the adsorption capacity of DNPH-γ-Al_2_O_3_ for Ni (II) was 18.18 (mg g^−1^) at pH = 5 and that the process followed the Langmuir isotherm [26]. In another study, the experimental data for the adsorption of Ni (II) on Fe_3_O_4_-GS revealed that the process followed the Langmuir isotherm and that the maximum adsorption capacity was 158.5 mg g^−1^ at pH = 6 [28]. The parameters of the pseudo-first-order and pseudo-second-order sorption kinetic models have been presented in Table 3. In order to evaluate the applicability of these kinetic models to fit the experimental data, K_1_ and K_2_ constants were determined experimentally from the slope and intercept of straight-line plots. The value of qe.c earned from the pseudo-second-order model was 28.25 mg g^−1^ for Cr (VI) and 25.97 mg g^−1^ for Ni (II) ions, which perfectly corresponded to the experimental values of qe (24.25 and 25.12 mg g^−1^) for Cr (VI) and Ni (II) ions. Overall, the pseudo-second-order model (R^2^: 0.9871 and 0.9947 % for Cr (VI) and Ni (II), respectively) was more efficient compared to the pseudo-first-order model (R^2^: 0.8422 and 0.8862 % for Cr (VI) and Ni (II), respectively). Because all the correlation coefficients were higher than 0.98 %, Cr (VI) and Ni (II) adsorption onto Fe_3_O_4_-NH_2_ might take place through a chemical process involving valence forces through sharing or exchange of electrons [25]. In another kinetic study using Fe_3_O_4_adsorbent, R^2^ value of Ni (II) was 0.998 at the optimum pH of 5.5. Therefore, the results showed that Ni (II) adsorption on Fe_3_O_4_ could be followed by the Freundlich model [29]. In order to measure the thermodynamic parameters for Cr (VI) and Ni (II) adsorption on Fe_3_O_4_-NH_2,_the adsorption studies were accomplished at 298.15 to 313.15 °K. The negative values of ΔG^θ^ at different temperatures, positive value of ΔS^θ^, and positive value of ΔH^θ^ during the adsorption of Cr (VI) and Ni (II) on Fe_3_O_4_-NH_2_ indicated that the adsorption was spontaneous, increased randomness at the solid-solution interface, and was endothermic in nature [18]. In addition, the slope and intercept of the plot of lnK vs. 1/T indicated the ΔH^θ^ and ΔS^θ^ values [18]. The values of standard enthalpy change (ΔH^θ^) and standard entropy change (ΔS^θ^), which were related to distribution coefficient (K_D_), were calculated and presented in Table 2. Using the ΔH^θ^ and ΔS^θ^ values, standard free energy changes (ΔG^θ^) for Fe_3_O_4_-NH_2_ were estimated. The results indicated that adsorption of Cr (VI) and Ni (II) on Fe_3_O_4_-NH_2_ could be followed spontaneously, was endothermic, and was entropy favored in nature. The positive value of ΔS^θ^ proved increase in the randomness at the solid-solution interface during the adsorption of Cr (VI) and Ni (II) on Fe_3_O_4_-NH_2_. This indicated that the amino-functionalized magnetic Nano-adsorbent (Fe_3_O_4_-NH_2_) could be regarded as an efficient and low cost adsorbent. The results of thermodynamic study in our research indicated that ΔG^ɵ^, ΔH^ɵ^, and ΔS^ɵ^ were respectively −3.28, 137.1, and 26.91 kJ mol^−1^ for Cr (VI) and −6.8433, 116.7, and 31.02 kJ mol^−1^ for Ni (II). Shen et al. conducted a similar study using adsorbent DETA-NMPs and disclosed that ΔG^ɵ^, ΔH^ɵ^, and ΔS^ɵ^ were −13.7, 8.41, and 72.83 kJ mol^−1^, respectively for Cr (VI) [25]. Hence, the results indicated that adsorption of Ni (II) on DETA-NMPs could be followed spontaneously, was endothermic, and was entropy favored in nature [25]. One other study also reported that ΔG^ɵ^, ΔH^ɵ^, and ΔS^ɵ^ were −1.599, 8.438, and 83.1, respectively for Ni (II) adsorption on Nano-HAP [30].

**Table 2:** The kinetic and thermodynamic and isotherm constants for the adsorption of Cr (VI) and Ni (II) by Fe_3_O_4_ NH_2_ and other adsorbents

Tem.	Adsorbent	pH		Pseudo-second-order			Thermodynamic parameters				Ref.
(k)		Cr (VI)	Ni (II)	K_2_ (g/mg^−1^) (min)^−1^	q_e,cal_ (mg/g^−1^)	R^2^	ΔG^ɵ^ (kJ mol^−1^)	ΔH^ɵ^ (kJmol^−1^)	ΔS^ɵ^ (J (mol K)^−1^)		
308	EDA-NMPs	2.5	-	0.7862	-	1	−1.61	10.59	37.50		[25]
308	DETA-NMPs	2.5	-	0.3668	-	1	−1.76	9.67	34.64		[25]
308	TETA- NMPs	2.5	-	0.3219	-	1	−2.15	23.15	76.82		[25]
308	TEPA- NMPs	2.5	-	0.1042	-	1	−2.36	10.46	38.04		[25]
303	Activated Alumina	3	-	-	0.0757	0.999	9.78	-	-		[40]
298	LewaitMP 610	5	-	-	-	-	−10.40	−2.51	35.49		[5]
298	Fe_3_O_4_	-	6	0.004		0.998	-	-	-		[41]
298	ZnO	-	6	0.002		0.998	-	-	-		[41]
298	CuO	-	6	0.019		0.995	-	-	-		[41]
303	Bagass Fly ash	5	-	-	-	-	−1.46	14.24	49		[42]
293	Nano-HAP	-	6.6	-	-	-	−1.599	8.438	83.1		[30]
298	Superparamagnetic Iron Oxide	-	5.5	-	-	-	27.9	7.8	110		[29]
293	Fe_3_O_4_-GS	2	-	0.055	17.29	0.999	−4.182	76.63	18.28		[28]
293	Fe_3_O_4_-GS	-	6	0.0203	22.07	0.998	−3.456	31.86	5.965		[28]
323	Fe_3_O_4_-TW	-	6	-	-	-	10.02	33.41	0.5799		[6]
298	Waste tea	-	4	-	-	-	−3.82	17.07	20.92		[43]
313	DETA-NMPs	-	6	1.03	9	0.999	−13.7	8.41	72.83		[25]
298	Fe_3_O_4_-NH_2_	3	-	0.002	28.25	0.987	−3.2891	137.1	ΔS^ɵ^	R^2^	This study
									26.91	0.975	
298	Fe_3_O_4_-NH_2_	-	6	0.008	25.97	0.994	−6.8433	116.7	31.02	0.960	This study
303	Fe_3_O_4_-NH_2_	Cr (VI)	Ni (II)	-	-	-	ΔG^ɵ^Cr (VI)		ΔG^ɵ^Ni (II)		This study
		3	6				−5.5038		−8.424		
308	Fe_3_O_4_-NH_2_	3	6	-	-	-	−9.7477		−10.045		This study
313	Fe_3_O_4_-NH_2_	3	6	-	-	-	−13.234		−12.934		This study
If: RL > 1, the adsorption is unfavorable. RL = 1, the adsorption is linear. 0 < RL < 1, the adsorption is favorable. RL = 0, the adsorption is irreversible.											[17, 44, 45]
If: 1/n < 1, the adsorption is unfavorable. If: 0.1 < 1/n < 1, the adsorption is favorable.											[46]
Tem.	Adsorbent	pH		Freundlich constants			Langmuir constants				Ref.
(k)		Cr (VI)	Ni (II)	K_F_ (Lg^−1^)	n	R^2^	q_max_ (mg g^−1^)	K_L_ (Lmg^−1^)		R^2^	
308	EDA-NMPs	2.5	-	-	-	-	136.98	0.1648		0.999	[25]
308	DETA-NMPs	2.5	-	-	-	-	149.25	0.4467		0.999	[25]
308	TETA- NMPs	2.5	-	-	-	-	204.08	0.075		0.998	[25]
308	TEPA- NMPs	2.5	-	-	-	-	370.37	0.1233		0.999	[25]
298	Walanut	3.5	-	0.244	3.36	0.989	8.01	2.98		0.964	[2]
298	Hazelnut	3.5	-	0.386	2.38	0.992	8.28	4.42		0.976	[2]
298	Almand Shells	3	-	0.153	2.68	0.984	3.40	0.580		0.972	[2]
303	Activated Alumina	3	-	2.84	1.80	09880	25.57	0.467		0.991	[40]
298	Mag	2.5	-	4.90	2.94	0.729	20.16	0.262		0.998	[47]
298	MagDt-H	2.5	-	1.62	2.63	0.984	13.88	0.030		0.965	[47]
298	Lewait MP 610	5	-	-	-	-	.41	-		0.99	[5]
298	PAC	-	8	0.02	2.85	0.708	31.08	0.27		0.98	[48]
298	Bagass	-	8	1.4E-03	0.868	0.868	0.03	0.95		0.97	[48]
298	Fly ash	-	8	2.03E-04	0.696	0.811	0.001	0.95		0.98	[48]
298	Fe_3_O_4_	-	6	1.550	0.996	-	-	-		-	[41]
298	ZnO	-	6	0.319	0.991	-	-	-		-	[41]
298	CuO	-	6	0.162	0.992	-	-	-		-	[41]
303	Bagass Fly ash	5	-	1.86	12.05	-	4.35	0.014		0.987	[42]
298	NH_2−_MCM-41	5	-	2.759	2.2	0.900	12.36	0.2245		0.956	[49]
293	Nano-HAP	-	6.6	8.87	2.74	0.934	46.17	0.07		0.995	[30]
298	DNPH-γ-Al_2_O_3_	-	5	1.95	2.037	0.926	18.18	1.426		0.985	[26]
293	Fe_3_O_4_-GS	2	-	30.58	3.01	0.997	39.92	4.08		0.959	[28]
293	Fe_3_O_4_-GS	-	6	3.801	2.56	0.993	158.5	0.2830		0.966	[28]
323	Fe_3_O_4_-TW	-	6	4.85	1.78	0.975	38.30	0.085		0.996	[6]
298	Superparamagnetic Iron Oxide	-	5.5	0.113	0.213	0.986	0.189 mmol/g	1.39 L/mmol		0.999	[29]
298	Waste tea	-	4	0.258	0.93	0.922	15.26	0.088		0.996	[43]
333	Fe_3_O_4_ -CNTs	-	2	7.23	3.05	0.981	65.96	0.42		0.997	[44]
313	DETA-NMPs	-	6	1.304	2.54	09026	43.24	0.288		0.999	[25]
298	Fe_3_O_4_-NH_2_	3	-	1.13	2.05	0.940	222.12	KL	RL	0.995	This study
								0.314	0.03-0.39		
298	Fe_3_O_4_-NH_2_	-	6	1.44	1.83	0.979	232.51	0.383	0.02-0.34	0.988	This study
If: RL > 1, the adsorption is unfavorable. RL = 1, the adsorption is linear. 0 < RL < 1, the adsorption is favorable. RL = 0, the adsorption is irreversible.											[17, 44, 45]
If: 1/n < 1, the adsorption is unfavorable. If: 0.1 < 1/n < 1, the adsorption is favorable.											[46]

**Table 3:** Kinetic adsorption parameters obtained using Pseudo-first-order and Pseudo-second-order models

Fe_3_O_4_-NH_2_	Pseudo-first-order				Pseudo-second-order		
Metals	K_1_ (min^−1^)	q_e,exp_ (mg/g^−1^)	q_e,cal_ (mg/g^−1^)	R^2^	K_2_ (g/mg^−1^) (min)^−1^	q_e,cal_ (mg/g^−1^)	R^2^
Cr (VI)	0.06	24.25	06.41	0.8422	0.002	28.25	0.9871
Ni (II)	0.03	25.12	14.64	0.8862	0.008	25.97	0.9947

Overall, simple preparation, fast separation, and high adsorption capacity of Fe_3_O_4_-NH_2_ make it a potential applicant for Cr (VI) and Ni (II) removal. Considering the larger adsorption capacity of Fe_3_O_4_-NH_2_ attributed to the amino-groups modified on the surface of Fe_3_O_4_-NH_2_, the amino-groups played a very important role in the adsorption process of Cr (VI) and Ni (II) in aqueous solution. This indicated that the increase of nitrogen percentage in Fe_3_O_4_-NH_2_ could result in an increase in the value of q_m_. Similar results were also obtained by Shen et al. [27] and Zhao et al. [25].

### Desorption and reusability of Fe_3_O_4_-NH_2_

**Fig. 8:**
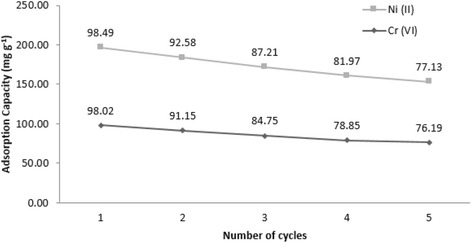
Adsorption and desorption efficiency of Cr (VI) and Ni (II) by Fe_3_O_4_-NH_2_ in adsorption—desorption cycle

For practical application of a cost-effective adsorbent for Cr (VI) and Ni (II) removal, desorption of metal ions from adsorbent and regeneration of Fe_3_O_4_-NH_2_ is of particular importance. Since the adsorption of Cr (VI) and Ni (II) onto Fe_3_O_4_-NH_2_ highly depends on the solution pH, desorption of the two heavy metals can be achieved by adjusting the pH. In the present study, the adsorption reversibility of Cr (VI)-laden Fe_3_O_4_-NH_2_ and Ni (II)-laden Fe_3_O_4_-NH_2_ was examined using NaOH (0.01, 0.05, 0.1, 0.2, and 0.3 mol L^−1^) and HNO_3_ (0.001, 0.005, 0.01, 0.05, and 0.1 mol L^−1^). For desorption studies, the metal-adsorbed modified Fe_3_O_4_ nanoparticles were first washed by ultrapure water for three times to remove the unadsorbed metals loosely appended to the adsorbent. When the concentration of NaOH and HNO_3_ was increased, the removal efficiency of desorption increased, as well. The best result was achieved with 2 min sonication in the presence of 0.2 mol L^−1^ NaOH and 0.05 mol L^−1^ HNO_3_. Finally, the Fe_3_O_4_-NH_2_ was dried in an oven (at 50 °C) during regeneration. In our study, each sorption/desorption process experienced a base and a heat treatment. The adsorption/desorption cycle results showed that the Fe_3_O_4_-NH_2_ could be reused for 5 times. Besides, the results presented in Fig. 8 indicated that at the end of the fifth cycle, the Fe_3_O_4_-NH_2_ maintained more than 76.19 % of its original Cr (VI) adsorption capacity and 77.13 % of its original Ni (II) adsorption capacity. Therefore, the great reusability Fe_3_O_4_-NH_2_ demonstrated its good potential for practical application.

### The effect of real water matrix

In this study, 1.0 and 5 mg L^−1^ Cr (VI) and Ni (II) were spiked with tap water and industrial wastewater for evaluating the practical application of Fe_3_O_4_-NH_2_. The initial concentration, pH, and removal efficiencies of Cr (VI) and Ni (II) after treatment with Fe_3_O_4_-NH_2_ have been presented in Table 4. According to the results, the removal efficiency of Cr (VI) at the concentration of 1.0 mg L^−1^ was 97.94 and 98.56 % for tap water and industrial wastewater, respectively. These measures were respectively obtained as 96.12 and 97.24 % for Ni (II) at the concentration of 1.0 mg L^−1^. This implies the excellent potential of Fe_3_O_4_-NH_2_ in water and wastewater treatment.

**Table 4:** The adsorption efficiencies of Cr (VI) and Ni (II) by Fe_3_O_4_-NH_2_ from tap water and industrial wastewater

Matrix	pH _Cr(VI)_and pH _Ni(II)_	Cr (VI) _initial_ and Ni (II) _initial_ (mg L^−1^)	Cr (VI) _removal_ (%)	Ni (II) _removal_ (%)
Tap water	6.6	1	97.94	98.56
Tap water	6.6	5	96.85	97.61
Industrial wastewater	6.2	1	96.12	97.74
Industrial wastewater	6.2	5	95.25	96.42

## Conclusion

In this study, Fe_3_O_4_-NH_2_ was prepared using a simple, cost-effective, and environmentally friendly method for the removal of Cr (VI) and Ni (II) ions from aqueous solution and was characterized by SEM, TEM, XRD, and VSM. The effects of controlling parameters, such as contact time, temperature, pH, Fe_3_O_4_-NH_2_ dose, and initial concentration of both heavy metals, were studied, as well. Based on the results, the Langmuir model fitted the isotherm data for both heavy metals and the maximum sorption capacity was 232.51 mg g^−1^ at pH = 3 for Cr (VI) and 222.12 mg g^−1^ at pH = 6 for Ni (II). Moreover, the adsorption kinetic data for Cr (VI) and Ni (II) were based on the assumption of a pseudo-second-order model and thermodynamic parameters showed that the adsorption process was endothermic, spontaneous, and entropy favored in nature. In addition, this nano-adsorbent was able to remove over 96 % of both heavy metals from tap water and industrial wastewater. The Fe_3_O_4_-NH_2_ could be regenerated with acid after adsorption and the adsorption capabilities only decreased with 6-7 % for both metal ions after five cycles. Overall, this study indicated that an amino-functionalized magnetic nano-adsorbent was promising for removal of Cr (VI) and Ni (II) ions in field application.

## Highlights

A sensitive method was developed for removal of Cr (VI) and Ni (II) from aqueous solution.

In-lab synthesized magnetic nanoadsorbent was developed by grafting amino-groups onto the surfaces of Fe_3_O_4_ nanoparticles.

The adsorbent was characterized by Transmission Electron Microscopy (TEM), powder X-Ray Diffraction (XRD), Vibrating Sample Magnetometry (VSM), and Scanning Electron Microscope (SEM).

The effects of pH, initial concentrations of Ni (II) and Cr (VI), adsorption kinetics, thermodynamics, and adsorption isotherm were studied.

## Abbreviations

SEM, scanning electron microscope; TEM, transmission electron microscopy; VSM, vibrating sample magnetometry; XRD, powder X-Ray diffraction
